# How to Apply Intraoperative Ultrasound when Spinal Trauma Surgery Is Performed in the Lateral Decubitus Position?

**DOI:** 10.1111/os.13953

**Published:** 2023-12-04

**Authors:** Bohan Li, Fayin Liu, Chunzheng Gao, Yong Qiao, Jie Zhao, Yang Song, Wen Xu, Kun Zhao, Chuanhong Dou, Hang Du, Xiaoqian Kong, Dongjin Wu

**Affiliations:** ^1^ Healthcare management center The Second Hospital of Shandong University Shandong People's Republic of China; ^2^ Zibo Hospital of Traditional Chinese Medicine Shandong People's Republic of China; ^3^ Department of Spine Surgery The Second Hospital of Shandong University Shandong People's Republic of China

**Keywords:** Decompression, Intraoperative Ultrasound, Lateral Decubitus Position, Posterior Approach, Spinal Trauma, Surgery

## Abstract

**Objective:**

At present, intraoperative ultrasound was widely used in spinal surgery. But there have been no reports on the use of intraoperative ultrasound in lateral decubitus position spinal surgery. The authors’ research objective was to describe the applications of intraoperative ultrasound in spinal trauma surgery when performed in the lateral decubitus position.

**Methods:**

Six patients with polytrauma who underwent surgery for spinal trauma between June 2020 and March 2022 and could not be operated on using a posterior approach in the prone position. All six patients underwent surgery in the lateral decubitus position. During surgery, a capsular bag had been designed and surgical field can be filled with normal saline for acoustic coupling, and then ultrasound was used to observe and guide decompression, and assess injuries of the neural elements such as the spinal cord. The data of preoperative and postoperative (12 months) American Spinal Injury Association impairment scale (AIS), follow‐up time, operation time, blood loss, ultrasound signal change of spinal cord, ultrasound guide decompression, internal fixation (12 months), and fracture healing(12 months) were collected.

**Results:**

The study included four males and two females whose ages ranged from 19 to 56 years old (41.5 ± 13.06 years old). Follow‐up times ranged from 12 to 20 months (14.33 ± 2.75 months). The operation times ranged from 195 to 248 mins (222.16 ± 16.86 mins). The estimated volume of blood loss ranged from 280 to 450 mL (383.33 ± 55.58 mL). The six cases’ AIS (preoperative vs. postoperative) were A versus A, C versus D, A versus B, B versus B, B versus C, and B versus C. Intraoperative ultrasound was performed successfully in all patients using our designed method. Intraoperative ultrasound observation revealed varying degrees of changes in spinal cord echo in all patients. Intraoperative ultrasound provided excellent assistance in spinal cord decompression during surgery. The surgery was completed successfully with no surgery‐related complications till the last follow‐up. At the time of last follow‐up (median time of 12 months) satisfactory fracture reduction and good internal fixation was confirmed on postoperative computed tomography scans and radiographs.

**Conclusions:**

The authors represented the technology of intraoperative ultrasound in spinal trauma surgery when performed in the lateral decubitus position. This technology solves how to apply intraoperative ultrasound in lateral decubitus position.

## Graphical abstract

To describe the applications of intraoperative ultrasound in spinal trauma surgery when performed in the side‐lying position without adding to the invasiveness of the procedure.

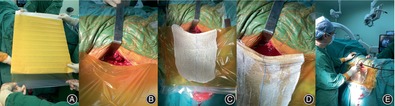

## Introduction

Intraoperative ultrasound is a useful tool during spinal surgery. It can observe the location and degree of spinal cord injury dynamically and in real time, guide intraoperative decompression, and observe whether decompression is complete, making diagnosis and surgery more accurate and precise. Nowadays, intraoperative ultrasound has become an important auxiliary tool in spinal surgery.^1^

Intraoperative ultrasound was used for the first time in surgery by researchers in the 1950s but was not used widely for cognitive and technical reasons, and until the 1980s, it had been widely accepted in spinal surgery.^2^, ^3^, ^4^ The intraoperative ultrasound technique is particularly useful for assessment of soft tissue structures, such as the intervertebral discs, intradural and epidural tumors, and spinal cord compression from the ventral side during spinal surgery. It can help surgeons to see important structures outside the surgical field of view, evaluate whether the spinal cord has been adequately decompressed, and decide whether a further decompression procedure is required.^5^

In some spinal trauma cases, it is impossible to judge whether decompression is sufficient under direct vision. Because the bone fragments displaced into the spinal canal are often located between the pedicles, these displaced bone fragments may not be visible on lateral fluoroscopy, and it is difficult to determine whether optimal reduction has been obtained. Furthermore, when using fluoroscopy, the ribs may obscure the thoracic spine and the shoulders obstruct spine view of the upper thoracic segments, which affects the surgeon's ability to judge whether the bone fragments displaced into the spinal canal are appropriately reduced. Furthermore, in obese patients, vertebral structures may not be clearly displayed during intraoperative fluoroscopy, making it impossible to judge whether the fracture is completely reduced and whether the dural sac is completely decompressed. Under these conditions, intraoperative ultrasound technology can be used to observe the ventral structure of the dural sac and to guide further intraoperative decompression.^6^

The ultrasound technique has been used in spinal surgery with the patient in the prone position.^7^ Water is used as a sound‐transmitting medium to detect the neural elements during surgery. In the prone position, the neural structures are at the bottom of the surgical field. After normal saline is added to the surgical field, the liquid just covers the dural sac. When spinal surgery is performed in the lateral decubitus position, the incision and deep neural structures are located at the same level, and the dural sac cannot be covered after addition of the normal saline. Therefore, using the traditional method, ultrasound cannot be used to explore the neural structures, assess whether compression from the ventral side of the dural sac is completely removed, and determine whether the fracture is correctly reduced, or evaluate injuries of the neural elements. Until now, there have been no reports on how to use ultrasound during spinal trauma surgery performed in the lateral decubitus position. This report on six thoracolumbar trauma cases is the first to demonstrate this technique when performed during spinal trauma surgery in the lateral decubitus position.

The purpose of this study was to (i) provide a simple, useful way of performing intraoperative ultrasound to ensure complete decompression of the spinal canal and (ii) to evaluate the status of the neural elements dynamically in real time during spinal trauma surgery in the lateral decubitus position without adding to the invasiveness of the procedure.

## Material and Methods

### Methods

The cases on this series were six patients who received treatment for multiple trauma at our institution between June 2020 and March 2022. All six patients could only undergo surgery via a posterior spinal approach in the lateral decubitus position instead of the prone position because of flail chest caused by multiple rib fractures on both sides, a pelvic fracture, injuries to multiple abdominal organs, and repair surgeries. A capsular bag was designed intraoperatively and constructed using the methods described in the following section. Intraoperative ultrasound was used to guide decompression and observe the injuries of the neural elements.

Inclusion criteria: (i) patients with polytrauma; (ii) patients underwent surgery for spinal trauma who could not be operated on using a posterior approach in the prone position.

Exclusion criteria: Patients with simple traumatic spinal fractures who can undergo prone position surgery.

### Technique

**FIGURE 1:**
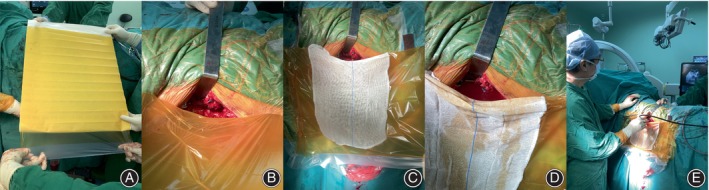
The manufacturing method of the bag containing normal saline for placing ultrasonic probe: another new antimicrobial incise drape was applied to cover the surgical area and adhered to the previously attached drape, with its upper edge ~3 cm higher than the incision, and a capsular bag (A, B) with an upward opening was made. The surgical area with no drape pasted was covered with sterile gauze to prevent the probe from adhering to it (C). The capsular bag and surgical field were filled with normal saline for acoustic coupling (D). A sterile 10‐MHz ultrasound probe was placed through the reserved window, and by scanning along the longitudinal axis and vertical to the body, sagittal and cross‐sectional images of the dural sac and neural elements were obtained (E).

The patients underwent posterior spinal surgery in the lateral decubitus position, during which the operating surgeon who has received ultrasound training used an 8862 ultrasound probe (BK Medical, Burlington, MA, USA). A posterior middle incision was made, and routine subperiosteal dissection of the paraspinal muscles was performed to expose the spinous processes, lamina, and facet joints on both sides; pedicle screws were implanted and connecting rods (Stryker, Inc. Kalamazoo, MI, USA) were installed. After the fractures were reduced using the screw‐rod system, the lamina at the level where the dural sac was compressed shown by magnetic resonance imaging was removed to expose the dural sac. After it was confirmed that the antimicrobial incise drape (3M Health Care, St. Paul, MN, USA) adhered before making the skin incision was correctly attached and the skin around the incision was not detached and waterproof, another antimicrobial incise drape was applied to cover the surgical area so that the upper edge of the drape was ~3 cm higher than the upper edge of the incision. The cephalad and caudal sides were pasted first, so that the reserved central part of the drape was 4–5 cm longer than the incision itself; next, the side of the antimicrobial incision drape was pasted well to the drape previously used before making the skin incision so that the two drapes were waterproof (Figure 1A,B). Then, the surgical area with no drape pasted was covered with sterile gauze to prevent the probe from adhering to it (Figure 1C), thus creating a capsular bag with the opening facing upward and being higher than the level of the surgical incision. The capsular bag and surgical field were filled with normal saline for acoustic coupling (Figure 1D). A sterile 10‐MHz ultrasound probe was placed through the reserved window, and by scanning along the longitudinal axis and vertical to the body, sagittal and cross‐sectional images of the dural sac and neural elements were obtained (Figure 1E). There have been several reports on how to use ultrasound during spinal surgery.^5^, ^8^ When the ultrasound probe is put into the normal saline, it is necessary to prevent it from coming into contact with the dural sac to avoid iatrogenic neural injury.

#### Outcome Measures

During surgery, ultrasound was used to observe and guide decompression, and assess injuries of the neural elements such as the spinal cord. The data of preoperative and postoperative (12 months) AIS, follow‐up time, ultrasound signal change of spinal cord, ultrasound guide decompression, internal fixation (12 months), and fracture healing (12 months) were collected.

## Results

There were four males and two females whose ages ranged from 19 to 56 years old (41.5 ± 13.06 years old). Follow‐up times ranged from 12 to 20 months (14.33 ± 2.75 months). The operation times ranged from 195 to 248 min (222.16 ± 16.86 min). The estimated volume of blood loss ranged from 280 to 450 mL (383.33 ± 55.58 mL). The six cases’ AIS (preoperative vs. postoperative) were A versus A, C versus D, A versus B, B versus B, B versus C, and B versus C. All six patients underwent surgery uneventfully. Intraoperative ultrasound was successfully used to explore decompression of the spinal canal and the injuries of the neural elements (Table 1). Postoperative imaging examination scans showed satisfactory reduction of the fracture segment and a good internal fixation position. The incision healed well postoperatively, with no surgery‐related complications till the final follow‐up.

**Table 1:** Surgical outcome data of six cases.

Case number	Gender	Age	Follow‐up time (months)	Operation time (min)	Blood loss (mL)	AIS		Ultrasound signal change of spinal cord	Ultrasound guide decompression	Internal fixation (12 months)	Fracture healing (12 months)
						Preoperative	Postoperative follow‐up (12 months)				
1	Male	52	12	248	450	A	A	Obvious swelling of the spinal cord at the level of the fracture, and heterogeneous mixture of slightly hyperechoic, isoechoic, and hypoechoic areas at the site of the spinal cord injury	Detect the spinal canal and confirm that reduction of the ventral bone of the dural sac was acceptable without obvious spinal cord compression	Excellent position	Yes
2	Female	19	12	195	280	C	D	The medullary cone and cauda equina were slightly swollen; the echo was still homogeneous and the cauda equina was shown to be herniating out of the dural sac	The displaced fracture segment was not well reduced and still compressing the cauda equina, then continue with decompression operation	Excellent position	Yes
3	Male	52	15	236	420	A	B	Swelling and thickening of the spinal cord at the fracture site, a large hypoechoic area in the spinal cord	Inoperative ultrasound found displaced bone segments compressing the spinal cord, then continue with decompression operation	Excellent position	Yes
4	Male	56	13	212	360	B	B	Significant spinal cord swelling, approximately twice the normal stage and a patchy hyperechoic area in spinal cord	Exploration revealed compression on the ventral side of the spinal cord and then continue with decompression operation	Excellent position	Yes
5	Male	36	14	220	420	B	C	Swelling and thickening of the spinal cord, the echo was still homogeneous	Reduction of the ventral bone of the dural sac was acceptable	Excellent position	Yes
6	Female	34	20	222	370	B	C	Heterogeneous mixture of slightly isoechoic and hypoechoic areas at the site of the spinal cord injury	Reduction of the ventral bone of the dural sac was acceptable	Excellent position	Yes

Abbreviation: AIS, association impairment scale.

### Typical Cases Presentations

#### Case 1

**FIGURE 2:**
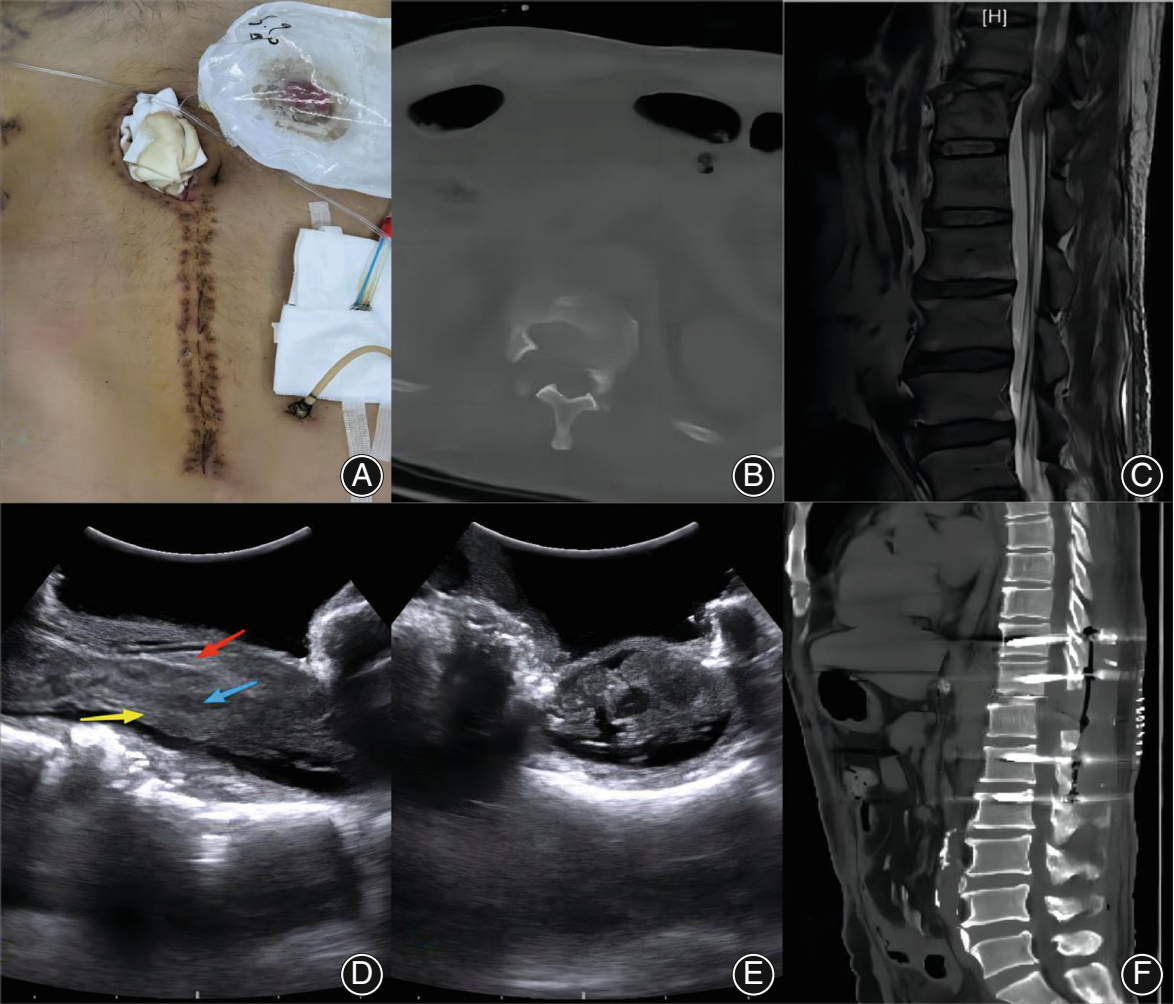
Case 1: The patient had pelvic fracture, enterostomy, and T‐tube drainage, and partial necrosis and dehiscence of the abdominal incision (A). Computed tomography (CT) and magnetic resonance imaging (MRI) showed comminuted fracture of T12 vertebral body and signal changes of spinal cord (B, C). The intraoperative ultrasound observation in lateral decubitus position showed that the spinal cord swelling and thickening were obvious (D), the injured spinal cord echo was heterogeneous (E), with slightly hyperechoic (D red arrow), isoechoic (D yellow arrow), and hypoechoic areas (D blue arrow). Postoperative CT showed satisfactory spinal canal decompression and reduction of fracture and good internal fixation position (F).

The patient was a 52‐year‐old man who sustained a spinal cord injury when he was hit by a falling weight. The diagnoses were as follows: hemorrhagic shock, thoracic (T11, T12) vertebral fractures, thoracic spinal cord injury with paraplegia (AIS A), pelvic fracture, multiple transverse process fractures of the lumbar vertebrae, liver and spleen contusions, left iliac vein injury, multiple bilateral rib fractures, and multiple soft tissue injuries. The patient had a pelvic fracture, enterostomy, and T‐tube drainage, and partial necrosis and dehiscence of the abdominal incision (Figure 2A). At a further multidisciplinary consultation, it was recommended not to take the prone position for spinal surgery, so the operation was performed in the lateral decubitus position. It was difficult to determine whether the displaced bone segment on the ventral side of the dural sac was appropriately reduced by intraoperative fluoroscopy. The laminectomy was performed at the level of canal encroachment, after which intraoperative ultrasound was used to detect the spinal canal and confirm that reduction of the ventral bone of the dural sac was acceptable without obvious spinal cord compression; there was obvious swelling of the spinal cord at the level of the fracture, and there was a heterogeneous mixture of slightly hyperechoic, isoechoic, and hypoechoic areas at the site of the spinal cord injury (Figure 2D,E). Postoperative computed tomography scans showed satisfactory reduction of the fracture segment and a good internal fixation position (Figure 2F).

#### Case 2

**FIGURE 3:**
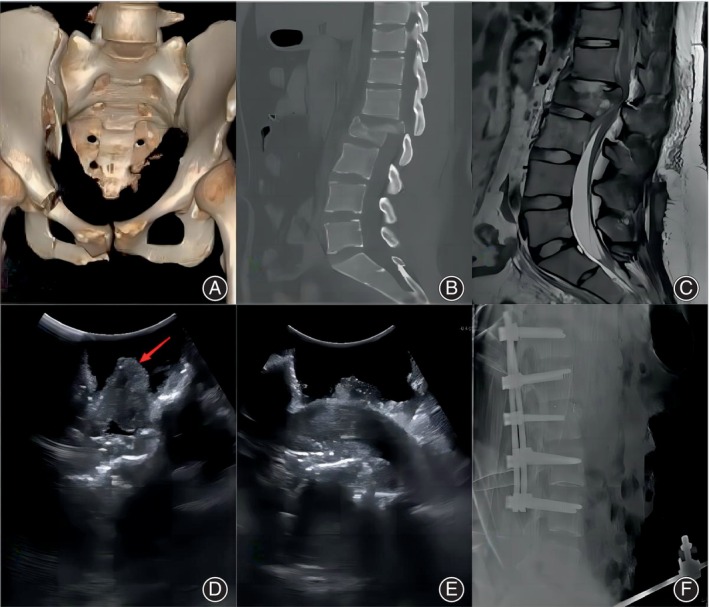
Case 2: Three‐dimensional computed tomography (CT) of pelvis showed unstable pelvic fractures (A). Spine CT and magnetic resonance imaging showed L2 burst fracture, and the fracture fragment encroach spinal canal compressing dural sac and cauda equina (B, C). In the lateral decubitus position, intraoperative ultrasound showed that cauda equina nerve herniated from the dural sac (D red arrow), the echo of conus medullaris and cauda equina was homogeneous. A subsequent ultrasound examination showed complete decompression of the spinal canal (E). Postoperative follow‐up radiographs showed satisfactory reduction of the fracture and a good internal fixation position (F).

The patient was a 19‐year‐old girl who was injured by a weight that fell from a height of ~10 m. The preoperative diagnoses were thoracic (T8, T9) vertebral compression fractures, a vertebral burst fracture at L2 with incomplete paralysis (AIS C), fractures of the spinous processes at L3 and L4 and the right transverse process at L1, L4, and L5, an unstable pelvic fracture, and a right lung contusion. After general anesthesia, external fixation with a fixator was performed first for the pelvic fracture, after which the patient was moved into the lateral decubitus position for reduction and fixation of the lumbar fracture, decompression of the spinal canal, and interbody fusion at L1/2 via a posterior approach. Intraoperative ultrasound was used to guide decompression and observe the injuries of the neural elements, as in the first case. The method used in case 1 was also used in case 2 to explore whether there was residual compression on the ventral side of the spinal canal and the injuries of the neural elements (Figure 3D). Ultrasonography revealed that the medullary cone and cauda equina were slightly swollen; however, the echo was still homogeneous and the cauda equina was shown to be herniating out of the dural sac. The displaced fracture segment on the ventral side of the dural sac was not well reduced and was still compressing the cauda equina. In this case, the facet joint on one side was resected to further reduce the displaced fragment and decompress the dural sac, after which a cage was implanted for fusion. A subsequent ultrasound examination showed complete decompression of the spinal canal (Figure 3E). Postoperative follow‐up radiographs showed satisfactory reduction of the fracture and a good internal fixation position (Figure 3F).

#### Case 3

**FIGURE 4:**
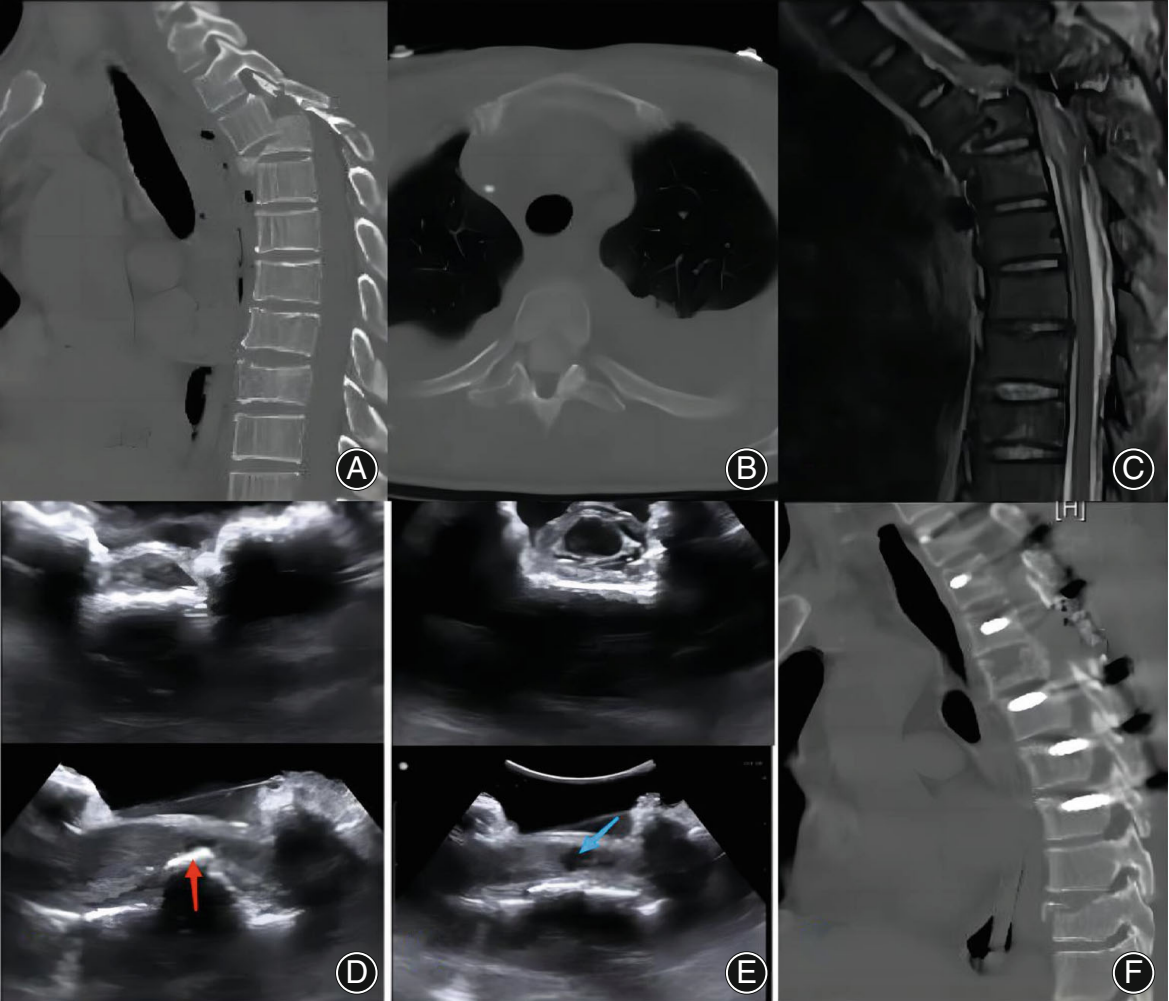
Case 3: Computed tomography (CT) and magnetic resonance imaging showed T3/4 fracture and dislocation, T9 compression fracture, and obvious spinal canal encroachment (A–C). The surgeon used intraoperative ultrasound in lateral decubitus position and observed swelling and thickening of the spinal cord at the fracture site, and found displaced bone segments compressing the spinal cord (D red arrow). After further decompression, it was observed that the spinal cord decompression was completed (E), and a large hypoechoic area (E, blue arrow) in the spinal cord was found. Postoperative CT showed satisfactory canal decompression and fracture reduction and good internal fixation position (F).

The patient was a 52‐year‐old man who was injured by a weight that had fallen from a height of ~9 m. The preoperative diagnoses were thoracic vertebral fractures (a burst fracture and dislocation at T4 and a compression fracture at T9), thoracic spinal cord injury with paraplegia (AIS A), right hemopneumothorax, multiple rib fractures and flail chest, effusion in the right thoracic cavity, cerebrovascular disease, scalp laceration, and a depressive state. The prone position was not recommended for spinal surgery after multidisciplinary consultation, so the surgery was performed in the lateral decubitus position. Intraoperative ultrasound was used to guide decompression and observe the injuries of the neural elements as in the previous cases. Intraoperative ultrasound revealed swelling and thickening of the spinal cord at the fracture site, a large hypoechoic area in the spinal cord, and displaced bone segments compressing the spinal cord (Figure 4D,E). Therefore, the facet joint was removed to achieve further decompression on the ventral side. The intraoperative ultrasound examination was repeated and showed complete decompression. Postoperative computed tomography scans confirmed satisfactory fracture reduction and a good internal fixation position.

## Discussion

In this study all six patients underwent surgery uneventfully. Intraoperative ultrasound was successfully used to explore decompression of the spinal canal and the injuries of the neural elements. The incision healed well postoperatively, with no surgery‐related complications till the final follow‐up.

### The Use of Intraoperative Ultrasound

When planning and implementing surgery for spinal trauma, it is important to determine the extent of injury, identify anatomic landmarks and any variants accurately, select a reasonable surgical approach, and ensure optimal decompression and rigid fixation. Therefore, more and more intraoperative imaging techniques are being developed for these operations. Intraoperative ultrasound technology has developed to the point that it can be used as an independent auxiliary method to obtain dynamic real‐time images, thereby ensuring safer and more accurate spinal surgery.^1^

Since the 1980s, intraoperative ultrasound has been widely used in spinal surgery and proved be helpful. In 1984, some researchers reported the feasibility of performing intraoperative ultrasonography in lumbar laminectomy and suggested that it had the advantage of being able to observe the spinal cord or cauda equina in the spinal canal, especially the structure on the ventral side of the dural sac, which could not be directly observed via a posterior surgical approach.^3^ A study in 1990 found that intraoperative ultrasound helped to ensure adequate bone decompression and complete discectomy.^9^ In the last 10 years, it has also been reported that intraoperative ultrasound could assess the amount of nerve root decompression and the degree of discectomy during surgery for thoracolumbar disc herniation.^10^, ^11^ There was a further report in 2011 of intraoperative ultrasound being used in intradural tumor surgery, which mentioned that some of its features were useful for differential diagnosis of various intradural tumors; furthermore, this technique also helped to improve surgical accuracy and could reduce the incidence of surgery‐related complications.^12^ In 2014, intraoperative ultrasound was reported to be a valuable tool for detecting spinal lesions, assessing surgical methods, developing surgical solutions, and observing the relationship between the location of lesions and bones, nerves, and blood vessels.^13^

In recent years, other spinal solutions, including the stereotactic navigation system, have become more precise in terms of anatomic localization, while intraoperative ultrasound is being improved in terms of resolution and remains an imaging method that can be used for dynamic real‐time monitoring of the neural elements. Furthermore, it can be used to observe the anatomic structure of soft tissues and bones around the dural sac, which is important when performing spinal decompression.^14^ A study in 2017 found that intraoperative ultrasound can detect whether the spinal cord is floating from the ossified posterior longitudinal ligament when assessing spinal cord decompression and that it is an effective method for assessment of indirect decompression of the posterior spinal cord with de‐kyphosis of thoracic spine.^15^ A study in 2022 found that intraoperative ultrasound was a useful tool in routine spine surgery and could simply and effectively detect decompression in a variety of pathologies, including herniated discs, epidural abscesses, tumors, deformities, and reconstructive surgery and that this simple tool was also helpful for surgical planning.^16^ The present report is the first to describe use of intraoperative ultrasound in surgery performed in the lateral decubitus position and shows that the echo characteristics and morphology of the spinal cord and cauda equina can be well displayed in this position (Figure 2D,E, 4D,E, 3D,E). Moreover, it demonstrated that intraoperative ultrasound could be used to guide decompression and to assess whether decompression on the ventral side of the dural sac was complete.

Intraoperative ultrasound is not only used to guide surgical decompression but also to determine the prognosis of certain diseases. A study published in 2021 found that use of intraoperative ultrasound allowed sufficient spinal cord expansion to be obtained after complete decompression in some but not all patients with cervical degenerative myelopathy. Insufficient expansion of the spinal cord after complete decompression heralded suboptimal neurological recovery in patients with degenerative cervical myelopathy.^17^ The authors of that report also mentioned that the ratio of the spinal cord central echo complex width to the anteroposterior diameter or transverse diameter of the spinal cord measured on transverse ultrasound images was associated with the prognosis of cervical spondylotic myelopathy.^18^ For the six patients with thoracolumbar fracture and dislocation described in this study, intraoperative ultrasound showed a correlation between changes in the echo images of neural elements and recovery of nerve function. Accumulation of more patients with traumatic spinal cord injury has shown that the extent of spinal cord lesions indicated by intraoperative ultrasound was not completely consistent with the extent of lesions indicated on magnetic resonance images. Therefore, some of the echo changes in the spinal cord indicated by intraoperative ultrasound might be better able to assess the prognosis of spinal cord injury, which will be discussed in detail in our separate article.

### Technology of this Study

Intraoperative ultrasound was a useful tool in routine spine surgery and could simply and effectively detect decompression of the surgery and that this simple tool was also helpful for surgical planning. In case 1, ultrasound was used to detect the spinal canal and confirm that reduction of the ventral bone of the dural sac was acceptable without obvious spinal cord compression. In case 2, ultrasonography revealed that the medullary cone and cauda equina were slightly swollen; however, the echo was still homogeneous and the cauda equina was shown to be herniating out of the dural sac. In case 3, intraoperative ultrasound revealed swelling and thickening of the spinal cord at the fracture site, a large hypoechoic area in the spinal cord, and displaced bone segments compressing the spinal cord. Therefore, the facet joint was removed to achieve further decompression on the ventral side.

The vast majority of traumatic spinal fracture patients can undergo prone position surgery, which allows surgeons to have a habitual surgical mindset. In this position, the surgeon can operate proficiently and obtain a very wide field of vision during decompression. According to the literature, the prone position should be used for all patients undergoing spine surgery guided by intraoperative ultrasound.^7^ In this position, the neural elements are located at the bottom of the surgical field. After normal saline is added, the liquid just covers the nerve structure, so that the surgeon can add normal saline directly into the surgical cavity for ultrasonic coupling to perform the intraoperative ultrasound examination. In the case of spinal surgery in the lateral decubitus position, the incision and deep neural structures are at the same level, and the dural sac cannot be covered after addition of normal saline. Therefore, the neural structures cannot be examined by an ultrasound probe to assess whether decompression of the dural sac is complete on the ventral side or whether ideal reduction of fracture dislocation has been achieved. When performing surgery in a lateral decubitus position, the surgeon needs to change their habitual thinking during the operation, and it was also inconvenient to perform decompression operations. So that intraoperative ultrasound was needed to determine whether complete decompression is necessary. This study was designed so that the antimicrobial incise drape could be used to construct a capsular bag, allowing intraoperative ultrasound technology to be used when performing spinal surgery in the lateral decubitus position.

The learning time of ultrasound technology in spinal surgery was relatively short, and spinal surgeons can apply intraoperative ultrasound after a short period of training. When applying intraoperative ultrasound, surgeons were required to always pay attention to the ultrasound screen in order to control the distance between the ultrasound probe and the spinal cord to avoid spinal cord injury.

### Strengths and Limitations

The method described in this paper could provide a solution to application of intraoperative ultrasound technology in spinal surgery performed in the lateral decubitus position in the future. When performing spinal surgery in a lateral position, intraoperative ultrasound technology can be used to guide the surgery and observe the ultrasound changes of the spinal cord, by using this method.

This series consists of only a small number of patients who underwent spinal surgery in the lateral decubitus position with use of intraoperative ultrasound. Currently, it is impossible to carry out a statistical comparison of intraoperative ultrasound in other patient positions in terms of factors such as operation time, intraoperative blood loss, and prognosis. However, we have successfully used ultrasound technology in patients undergoing spinal trauma surgery in a lateral decubitus position and found that it was valuable to guide surgical decompression.

## Conclusions

We have successfully used an ultrasound technique during spinal trauma surgery performed via a posterior approach in a lateral decubitus position, effectively solving the problem that intraoperative ultrasound imaging technique could not be applied in spinal surgery in lateral decubitus position.

## Author Contributions

All authors contributed to the article's conception and design. Dongjin Wu and Chunzheng Gao decided on the content. Yong Qiao, Jie Zhao, Xiaoqian Kong, and Kun Zhao were the guarantors of the overall preoperative data. Yang Song, Wen Xu, Hang Du, and Chuanhong Dou were the guarantors of the overall postoperative data. Bohan Li and Fayin Liu wrote the first draft of the manuscript. Dongjin Wu revised the manuscript. All authors have read and approved the final submitted manuscript.

## Conflict of Interest

The authors declare that there are no conflict of interest.

## Funding Statement

No funds were received in support of this work. No benefits in any form have been or will be received from a commercial party related directly or indirectly to the subject of this manuscript. The manuscript does not contain information about medical device(s)/drug(s).

## Ethics Statement

The study was approved by our institutional ethics committee, and the approval number is KYLL‐2022LW153. Informed consent was obtained from all patients in this series.

## References

[1] Tat J , Tat J , Yoon S , Yee AJM , Larouche J . Intraoperative ultrasound in spine decompression surgery: a systematic review. Spine. 2022;47(2):E73–E85. 34474449 10.1097/BRS.0000000000004111

[2] French LA , Wild JJ , Neal D . The experimental application of ultrasonics to the localization of brain tumors; preliminary report. J Neurosurg. 1951;8:198–203. 14824982 10.3171/jns.1951.8.2.0198

[3] Gooding GA , Boggan JE , Weinstein PR . Intraoperative sonography during lumbar laminectomy: work in progress. Am J Neuroradiol. 1984;5(6):751–753. 6437177 PMC8333652

[4] Vincent KA , Benson DR , McGahan JP . Intraoperative ultrasonography for reduction of thoracolumbar burst fractures. Spine. 1989;14(4):387–390. 2655113 10.1097/00007632-198904000-00007

[5] Ganau M , Syrmos N , Martin AR , Jiang F , Fehlings MG . Intraoperative ultrasound in spine surgery: history, current applications, future developments. Quant Imaging Med Surg. 2018;8:261–267. 29774179 10.21037/qims.2018.04.02 PMC5941206

[6] Lerch K , Völk M , Heers G , Bae W , Nerlich M . Ultrasound‐guided decompression of the spinal canal in traumatic stenosis. Ultrasound‐Guided Decompression of the Spinal Canal in Traumatic stenosisUltrasound in Medicine & Biology. 2002;28(1):27–32. 10.1016/s0301-5629(01)00489-6 11879949

[7] Chryssikos T , Wessell A , Pratt N , Cannarsa G , Sharma A , Olexa J , et al. Enhanced safety of pedicle subtraction osteotomy using intraoperative ultrasound. World Neurosurg. 2021;152:e523–e531. 34098140 10.1016/j.wneu.2021.05.120

[8] Vasudeva VS , Abd‐El‐Barr M , Pompeu YA , et al. Use of intraoperative ultrasound during spinal surgery. Glob Spine J. 2017;7:648–656. 10.1177/2192568217700100 PMC5624373 28989844

[9] Montalvo BM , Quencer RM , Brown MD , Sklar E , Post MJ , Eismont F , et al. Lumbar disk herniation and canal stenosis: value of intraoperative sonography in diagnosis and surgical management. AJR Am J Roentgenology. 1990;154(4):821–830. 10.2214/ajr.154.4.2107683 2107683

[10] Aoyama T , Hida K , Akino M , Yano S , Iwasaki Y . Detection of residual disc hernia material and confirmation of nerve root decompression at lumbar disc herniation surgery by intraoperative ultrasound. Ultrasound Med Biol. 2009;35(6):920–927. 19376637 10.1016/j.ultrasmedbio.2008.12.014

[11] Nishimura Y , Thani NB , Tochigi S , Ahn H , Ginsberg HJ . Thoracic discectomy by posterior pedicle‐sparing, transfacet approach with real‐time intraoperative ultrasonography: clinical article. J Neurosurg Spine. 2014;21(4):568–576. 25036220 10.3171/2014.6.SPINE13682

[12] Zhou H , Miller D , Schulte DM , Benes L , Bozinov O , Sure U , et al. Intraoperative ultrasound assistance in treatment of intradural spinal tumours. Clin Neurol Neurosurg. 2011;113(7):531–537. 21507563 10.1016/j.clineuro.2011.03.006

[13] Prada F , Vetrano IG , Filippini A , del Bene M , Perin A , Casali C , et al. Intraoperative ultrasound in spinal tumor surgery. J Ultrasound. 2014;17(3):195–202. 25177392 10.1007/s40477-014-0102-9 PMC4142127

[14] Alaqeel A , Abou Al‐Shaar H , Alaqeel A , et al. The utility of ultrasound for surgical spinal decompression. Med Ultrason. 2015;17(2):211–218. 26052573 10.11152/mu.2013.2066.172.spd

[15] Ando K , Imagama S , Ito Z , Kobayashi K , Ukai J , Muramoto A , et al. Ponte osteotomy during dekyphosis for indirect posterior decompression with ossification of the posterior longitudinal ligament of the thoracic spine. Clin Spine Surg. 2017;30(4):E358–E362. 28437338 10.1097/BSD.0000000000000188

[16] Rustagi T , Das K , Chhabra HS . Revisiting the role of intraoperative ultrasound in spine surgery for extradural pathologies: review and clinical usage. World Neurosurg. 2022;164:118–127. 35504481 10.1016/j.wneu.2022.04.116

[17] Chen G , Wei F , Shi L , Li J , Wang X , Wang M , et al. Inadequate spinal cord expansion in intraoperative ultrasound after decompression may predict neurological recovery of degenerative cervical myelopathy. Eur Radiol. 2021;31(11):8478–8487. 33929570 10.1007/s00330-021-08000-x

[18] Chen G , Wei F , Shi L , et al. Potential of intraoperative ultrasonographic assessment of the spinal cord central echo complex in predicting postoperative neurological recovery of degenerative cervical myelopathy.European. J Neurol. 2022;29(1):217–224. 10.1111/ene.15109 34528341

